# Everybody's Page

**Published:** 1891-02-28

**Authors:** 


### Everybody's Page.

The foundation by the late Sir William Gull of the Gull Studentship in Pathology, at the Medical School of Guy's Hospital, of the yearly value of £125, with an additional £25 for apparatus, is a practical way of commemorating the founder's fondness for that study which will commend itself to many future students.

The present difficulty, amounting often to impossibility, in the way of the peasants in Russia attending mass has been overcome in those sparsely inhabited tracts of country which are crossed by a railway by the novelty of introducing a travelling Church capable of seating seventy persons and performing parochial duties at several stations during the day.

The introduction by the Chief Commissioner of the City Police of the regulation permitting the constables on duty at night to go to the station of their division in the middle of the night in order to get a brief rest and some hot coffee will prove a great boon during cold winters such as the present, and doubtless will save great waste of strength. It will be interesting to compare the returns of the number of men in hospital for diseases of the respiratory organs before and after the introduction of this regulation.

The following story may commend itself as an eminently practical way out of an unpleasant and distasteful dilemma to those duellists (not in this country where duelling is obsolete) in France and other countries, who are not particularly anxious to exchange the certainties of this world, however destitute of happiness, for the great uncertainty of the next. Humphrey Howarth, the surgeon, when called out on a certain occasion to fight a duel made his appearance on the field stark naked, to the astonishment of his challenger, who asked him what he meant. "I know," said Howarth, "that if any of the clothing is carried into the body by a gunshot wound festering ensues, and, therefore, I have met you thus." His antagonist declared that fighting with a man in a state of nature would be quite ridiculous, and accordingly they parted without further discussion.

We are constantly hearing of people, with or without brains, in search of employment, yet there is evidently plenty of money to be made by the exercise of a little ingenuity. A certain showman is stated by the police to have attracted such crowds in East-end thoroughfares to view his *novelties* that he has earned as much as £16 in one night. The novelties consist of such attractive items as the white-eyed Kaffir, the talking donkey, and other delectables of a questionable and less refined nature. It would, however, appear advisable for the embryo showman in search of fame and pelf to avoid including amongst his stock dancing Zulus covered with jangling bells, as likely to create a difference of opinion amongst the admiring crowd as to the merits of the show. A showman, whose wares were evidently so popular as to be so productive, must have felt hurt at being summoned as an "abominable nuisance."

John Hunter believed that when there was only one daughter and several sons in a family, the daughter was always of a masculine disposition, and that when a family consisted of several daughters and only one son, the son was always effeminate. No doubt the experience of many parents will lead them to share this belief.

It is stated that at Leaky, in Lincolnshire, during the recent frost, the postman, taking advantage of the ice-covered state of the roads, delivered the whole of his letters on skates. His round measures eight miles, and over every inch of this he was able to skate without a break, thus combining pleasure with his business. How many footsore fellow-postmen would have envied him had they known of his good fortune!

Malta, which has presented statesmen with more than one nut to crack, has been kinder, it would appear, to naturalists. This charming island is stated to possess but one snake, and that not of a venomous order. This, in our eyes, adds considerably to the charms of Malta as a dwelling place, which, though outdistanced in the matter of snakes by another island up north, beats its rival in the matter of climate. A curious feature, and not altogether a pleasant one in this snake, is its power of holding on by its teeth, an innocent pastime which we believe is denied to its poisonous brethren.

The present generation, whose sanitary surroundings are a paradise compared with the state of affairs existing half a-century ago, cannot probably appreciate to the full what advance has been made since 1840, though they can see more clearly than their forefathers did that preventive medicine is a necessary part of the outfit of every practitioner, and that the authorities who fail in their duties in the administration of the sanitary laws are among the very worst oppressors of the poor. People are beginning to see that the only sure way in which to cope with drunkenness and crime is to acknowledge their responsibility in the matter of local Government and act up to it by improving the conditions under which the poorer members of society live.

The returns of the Registrar-General will be found to give some curious and interesting information as to the relative healthiness of occupations of the people in this country. Clergyman and farmers appear to be blessed with the longest lives, whereas amongst chimney sweeps, who suffer to a remarkable extent from cancer; file makers, bookbinders and printers, and all who work in a horrible atmosphere mortality runs very high. That inn-keepers and brewers should not be long lived will easily be understood, and there are many who predicted the evils which would arise from giving to grocers the right of dealing in wines and spirits, who will not be surprised to learn that since the extension to them of that right the mortality amongst them has risen considerably. Proximity to stimulants seems to have a baneful fascination which few can resist.

